# Mobility, mental well-being and Neighborhood walkability among older adults in Nigeria: an urban perspective

**DOI:** 10.1186/s13690-025-01722-0

**Published:** 2025-10-31

**Authors:** Jeneviv Nene John, Fredrick Sunday Isaac, Emmanuel Jonah Osiri, Ukamaka Gloria Mgbeojedo, Obinna Chinedu Okezue, Chimdi Ebubechi Nnamani, Sam Chidi Ibeneme

**Affiliations:** 1 Department of Medical Rehablitation, Faculty of Health Sciences and Technology, University of Nigeria, Enugu Campus, Enugu, Enugu State Nigeria; 2 Department of Physiotherapy, Federal Medical Centre, Umuahia, Abia State Nigeria; 3 Department of Physiotherapy, Faculty of Health Sciences, David Umahi Federal University of Health Sciences, Uburu, Ebonyi State Nigeria; 4 Department of Physiotherapy, Faculty of Health Sciences, School of Therapeutic Studies, University of the Witwatersrand, 7 York Road, Parktown, Johannesburg, Gauteng 2193 South Africa

**Keywords:** Mobility, Mental well-being, Neighbourhood characteristics, Older adults

## Abstract

**Background:**

The rapid urbanization and demographic shifts experienced globally have significant implications for the well-being of older adults, particularly in low and middle income countries like Nigeria. As individuals age, understanding the impact of environmental characteristics on mobility and mental well-being becomes crucial for optimal health. Walkable neighborhoods have the potential to enhance mobility, social engagement, and well-being among older adults. This study explored the neighborhood characteristics and its relationship with mobility and mental well-being of older adults in Nigeria. It also identified the policy implications of the findings for urban planning and public health interventions.

**Methods:**

This cross-sectional study involved 162 older adults residing in Enugu urban area between August 2023 and April 2024. The Neighborhood Environment Walkability Scale, Life Space Questionnaire and Warwick-Edinburgh Mental Well-being Scale assessed the neighborhood characteristics, mobility and mental well-being respectively. Correlation and multiple regression analysis were employed.

**Results:**

Significant relationships were observed between mobility and residential density (*r*= -0.223, *p* = 0.004), connectivity (*r* = 0.266, *p* = 0.001) aesthetics (*r* = 0.212, *p* = 0.007), and safety from traffic (*r* = 0.214, *p* = 0.006). The regression analysis with mobility as the dependent variable showed a good model fit and was significant [F = 7.389, *p* < 0.001, adjusted R^2^ = 0.28, R^2^ = 0.33]. The predictors/independent variables in the model accounted for 33% of the variance in mobility. Durbin Watson’s score was 2.066 indicating independence of observations in the model. Variance Inflation factor values were less than 10 while Tolerance values were greater than 0.20 indicating no multicollinearity in the model. Key predictors of mobility were residential density, land use mix, connectivity, aesthetics, safety from traffic, environmental description and child safety. The stepwise regression identified five significant predictors: connectivity, residential density, aesthetics, child safety, and traffic safety. These explained 27.5% of the variance in mobility scores (R² = 0.275, *p* < 0.001). While explaining slightly less variance than the full model, it offered a more interpretable structure. Interestingly, there was no significant relationship between neighborhood characteristics and mental well-being (*p* > 0.05), and none of these characteristics emerged as significant predictors of mental well-being.

**Conclusions:**

Urban planners, policymakers, and healthcare professionals should collaborate to create age-friendly environments that prioritize mobility and well-being for older adults. The insights from this study can inform policy recommendations and guidelines aimed at enhancing the overall quality of life for older adults, reducing the incidence of isolation, anxiety, and depression, and promoting healthier, more vibrant communities.

**Clinical trial number:**

Not applicable.

**Table:** 

Text box 1. Contributions to the literature
• Evidence on how neighborhood environments shape mobility and mental well-being in older adults is limited in sub-Saharan Africa; this study provides novel insights from a rapidly urbanizing Nigerian city.
• While some neighborhood characteristics predicted mobility, none were linked to mental well-being, indicating that broader socio-cultural or systemic factors may shape older adults' mental health.
• Offers critical insights for urban planning by emphasizing the public health importance of context-specific, age-friendly urban design to promote mobility and healthy aging.
• Supports the need for integrated urban policies that address the physical and social environments of aging populations in rapidly urbanizing regions.

## Background

Urbanization and population aging are two critical global demographic trends, with profound implications for the well-being of older adults, particularly in low- and middle-income countries like Nigeria [1, 2]. The United Nations [3] projects that the global population aged 65 years and above will rise from 9% in 2020 to 16% by 2050. While increased longevity signifies a triumph of development, it also demands strategies to address its societal impacts. Active aging is defined by the World Health Organization as the process of optimizing opportunities for health, participation, and security to enhance quality of life as people age [4]. In Nigeria, population ageing and rapid urbanization pose significant challenges to the well-being of older adults particularly in low and middle income countries [5]. Although Africa’s projected urbanization level of 64% by 2050 remains lower than that of more urbanized regions such as North America (82%), Latin America and the Caribbean (81%), and Europe (74%), the continent is urbanizing at an unprecedented pace [6]. This projected increase surpasses the global average urbanization rate of 56% as of 2021, highlighting Africa’s rapid urban transformation despite its comparatively lower baseline [6]. This trend underscores the urgent need to address the associated challenges and opportunities through effective urban planning and policy interventions. During this period of urbanization, Nigeria, India, and China are expected to account for 37% of global population growth [7]. The World Health Organization [7] highlights that mental health issues tend to intensify with age, leading to higher suicide rates among older adults compared to younger populations. Aging is often accompanied by a reduction in daily activity spaces and diminished social interactions [8–10], both of which can restrict mobility and contribute to social isolation. This decline in mobility adversely affects mental well-being, as physical activity and social engagement are essential for maintaining mental health [11, 12].

The neighborhood environment is crucial in shaping the health outcomes of older adults [13, 14]. Walkable neighborhoods characterized by safe, accessible pedestrian pathways and proximity to destinations facilitate mobility and reduce sedentary behavior [15, 16]. These environments provide opportunities for physical activity, leisure, and social participation, all of which enhance the quality of life for older adults [17]. Beard and Petitot [1] and Cerin et al. [2] emphasize that well-designed urban environments are essential for maintaining older adults’ independence and social connections. Furthermore, walkable neighborhoods encourage physical activity, as they tend to be densely populated, interconnected, and located near amenities like shops, parks, and public transport [18, 19]. Given that older adults often prefer walking within their local environments [2, 15, 17], enhancing neighborhood walkability is a critical consideration for promoting active aging.

Mobility, defined as the ability to move independently using assistive devices or transportation within and beyond one’s immediate environment [20], is integral to active aging. It supports independence, physical activity, social participation, and access to resources, which collectively improve physical and mental well-being [7, 21]. A supportive neighborhood environment that enhances mobility can minimize the risk of falls, injuries, and institutionalization, while promoting independence and quality of life in old age [22].

Neighborhood characteristics also reportedly influence psychological well-being. Structural and social features of the built environment can significantly affect mental health, as evidenced by studies [23, 24]. Adverse perceptions of neighborhood such as limited accessibility to green spaces, high crime rates, and low walkability are associated with depression, anxiety, and stress [9, 10, 25–27]. Conversely, favorable perceptions are linked to increased physical activity, which can mitigate depression and cardiovascular risks, benefiting both physical and mental health [28].

While extensive research has explored the relationships between neighborhood environments, mobility, and mental well-being in various contexts [29–32], there remains a gap in understanding these dynamics in Nigeria. Existing studies in Nigeria primarily focus on the influence of neighborhood environments on older adults’ quality of life and physical activity [33]. Also, Oyeyemi et al. [34] examined the association between neighborhood environmental factors and health-related moderate-to-vigorous physical activity, including walking for transportation and recreation, among community-dwelling Nigerian older adults. Other studies in Nigeria have been focused on the assessment of walkability constraints in the residential neighbourhoods of Enugu Metropolis, Nigeria [35] and on the measurement of neighbourhood walkability in Enugu, Nigeria [36]. However, there is no research addressing the interplay between neighborhood characteristics, mobility, and mental well-being in Nigeria.

Nigeria faces unique challenges associated with rapid urbanization and population aging, including disparities between population growth and economic development [37]. These challenges are exacerbated by inadequate healthcare infrastructure, limited social security systems, and insufficient urban planning, which increase older adults’ vulnerabilities [5, 38]. Thus, there is an urgent need for research tailored to Nigeria’s context to inform policies and interventions that create supportive neighborhoods, enhance mobility, and improve mental well-being for older adults in urban settings. Therefore, the aim of this study is to explore the relationship of neighborhood characteristics with mobility and mental well-being among older adults in an urban Nigerian context. Additionally, this study seeks to identify policy and urban planning implications arising from these relationships, with a view to informing evidence-based interventions for active and healthy aging.

## Methods

### Design

**Fig. 1:**
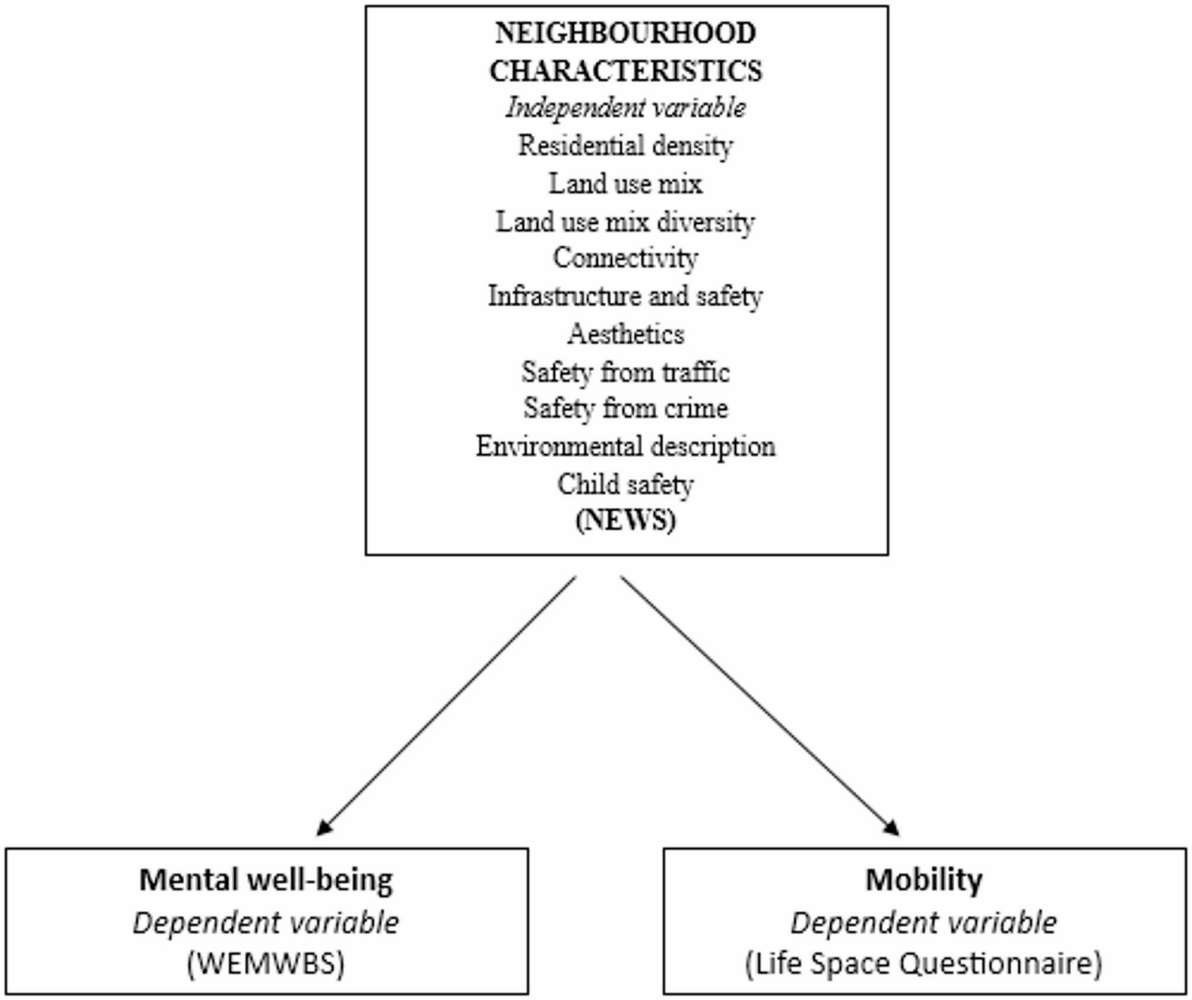
Conceptual framework illustrating the hypothesized relationships between perceived neighborhood characteristics, mobility, and mental well-being among older adults in Enugu, Nigeria (August 2023 – April 2024). **Legend**: Neighbourhood characteristics measured using the Neighbourhood Environment Walkability Scale (NEWS); Mental well-being measured using Warwick Edinburgh Mental Well-being Scale (WEMWBS); Mobility measured using the Life-Space Questionnaire (LSQ)

This study adopted a cross-sectional quantitative design to explore the relationship of neighbourhood characteristics with mobility and mental well-being among older adults. The conceptual framework in Fig. 1 reflects the organization of key variables in the study and the hypothesized direction of their relationships.

### Setting

The study was conducted across residential neighborhoods in Enugu urban area between August, 2023 and April, 2024. Enugu, the capital city of Enugu State, is located in the southeastern geopolitical zone of Nigeria and comprises three local government areas (LGA): Enugu East LGA, Enugu North LGA, and Enugu South LGA. It is recognized as one of the major urban centers in the country and is characterized by a mixed residential, commercial, and administrative land use [39, 40]. The population of Enugu State is projected to reach approximately 4.9 million by 2025, reflecting an estimated growth rate of about 3.5% from the 2022 population figure of 4,690,100 [41]. Enugu has a rapidly growing population of older adults and has been identified as an urban area undergoing increased urbanization and associated environmental transitions in Nigeria [5, 37]. Enugu exhibits a diverse residential landscape with varying population densities [36]. The present-day Enugu urban metropolis consists of eighteen formal and seven informal residential neighbourhoods [36]. Specifically, four of these residential neighborhoods are located in Enugu East LGA, seven in Enugu North LGA, and eight in Enugu South LGA [36]. These residential neighborhoods served as the primary units of recruitment for this study.

### Participants

The target population included older adults aged 60 years and above in residential neighbourhoods of Enugu urban metropolis. Using the sample size calculation for a cross-sectional study $\:N$= Z_1−&/2_^2^ p(1- p)/d^2^ with the absolute error between 4% and 5%, and the proportion of older adults at 4.78% [42], the researcher estimated that the appropriate sample size for this study will be between 73 and 114 [40]. This formula was applied to estimate a population proportion with a defined level of precision. Although the primary aim was not to detect a hypothesized effect size, the final sample size of 162 exceeds this minimum and provides sufficient power to detect small-to-moderate effect sizes (Cohen’s h ≈ 0.20) in group comparisons or associations, assuming a 95% confidence level and typical assumptions used in observational studies [43]. A stratified random sampling technique was utilized for this study. The three LGA within the Enugu urban metropolis, along with their respective residential settlements, were listed. All 25 residential neighbourhoods (18 formal and 7 informal) within each LGA were stratified into low, medium, and high density categories based on population density data from municipal records [36]. This stratification ensured diversity in both physical and social environmental features ensuring a robust representation of urban older adults. By applying a stratified random sampling technique, each population density category (low, medium, and high) had a proportional representation of participants, reflecting the different challenges and opportunities related to walkability and mobility that might exist across neighborhoods of varying densities. Older adults residing in these neighbourhods were identified and compiled using voter registers, health records, and community listings. Within each neighborhood density stratum, participants were selected using simple random sampling to ensure an unbiased and representative sample.

Of the 200 individuals selected, 192 expressed interests in participating. However, only 186 attended the follow-up screening as scheduled. During the screening, a geriatric doctor conducted assessments to confirm their eligibility. These geriatric assessments provided additional confirmation of the intended participants’ health status. Ultimately, this study included a total of 162 participants, exceeding the required sample size and ensuring sufficient power to detect statistically significant effects. Of the 162 participants included in the study, 52 were drawn from low density neighbourhoods, 56 from medium density neighbourhoods, and 54 from high density neighbourhoods. This near-equal distribution across the three density strata represents a balanced allocation that ensures adequate representation of each stratum, thereby reducing sampling bias and enhancing the internal validity of the study. The slight variation in numbers reflects the natural differences in the availability and eligibility of older adults within each density category, based on existing community records, voter registers, and health documentation. In some instances, a higher number of eligible individuals were identified in certain strata, particularly in medium-density areas. Furthermore, minor differences in response rates and availability during recruitment contributed to the final distribution. Participants eligible for this study were those aged 60 years or older, capable of ambulating independently without assistance, and cognitively intact. They must have resided in their current neighborhood for at least 12 months to ensure familiarity with the local environment and walkability features. Participants were excluded from the study if they presented with significant sensory impairments, such as severe hearing or vision loss, that could interfere with their ability to complete self-report measures reliably. Exclusion also applied to individuals with mobility-restricting conditions, such as recent fractures, advanced arthritis, or other medical conditions that severely limit physical activity or ambulation, as these could affect their experience and perception of neighborhood walkability. A history of recent cardiovascular events within the past six months, was another criterion, given the potential impact on their ability to safely engage in mobility-related activities. Chronic health conditions, such as uncontrolled hypertension, epilepsy, or other conditions that pose safety risks or hinder participation in mobility-related activities, were also grounds for exclusion. Additionally, individuals with severe psychological or neurological disorders that could impair their ability to engage with self-report measures were excluded.

### Instruments for data collection

The Nigerian version of the Neighborhood Environment Walkability Scale (NEWS) [44] was used to assess the participants perceived neighborhood characteristics. This validated instrument includes items across subscales such as residential density (types of residencies in the neighbourhood- 6 items), land use mix diversity (stores, facilities and other things in the neighbourhood − 28 items), land use mix (access to services − 5 items), connectivity (streets in the neighbourhood − 3 items), infrastructure and safety (places for walking and cycling- 18 items), aesthetics (neighbourhood surroundings − 6 items), traffic safety (7 items), safety from crime (4 items), additional environmental features (6 items which include parking being difficult in local shopping areas; the streets in my neighborhood being hilly, which makes it difficult to walk; the streets not having many cul-de-sacs, closes, or dead-end streets; the presence of canyons or hillsides that limit the number of routes for getting from place to place; opportunities to see and speak to other people while walking; and the presence of unattended or stray domestic animals in the neighborhood) and child safety (4 items). Oyeyemi et al. [44] indicated an acceptable test-retest reliability, construct validity, and a low-to-moderate concurrent validity for subscales of NEWS Nigeria. The test- retest reliability values (ICC = 0.59 to 0.91) reported for the NEWS subscales were comparable to those reported for the original version in the United States of America (ICC = 0.58 to 0.80) [45], the Australian version (ICC = 0.62 to 0.88) [46], and the Chinese translation (ICC = 0.57 to 0.99 [47].

Mobility was evaluated using the Life Space Questionnaire (LSQ), a tool validated for measuring spatial mobility among older adults [48]. The scores range from 0 (bedbound) to 120 (frequent independent travel outside the city). The LSQ has demonstrated strong psychometric properties in previous studies. Specifically, it has shown good construct and criterion validity for measuring the spatial extent of mobility in community-dwelling older adults [48, 49]. The LSQ exhibits good test-retest reliability (kappa coefficient = 0.80), strong criterion validity, and robust construct [48].

The Warwick Edinburgh Mental Well-being Scale (WEMWBS) utilized in previous research [50–52] was employed to assess the participants mental well-being. This scale was developed for the evaluation of mental wellbeing and was designed to measure a broad conception of mental well-being, including positive affect, psychological functioning, and interpersonal relationships. Respondents rate each of the 14 items on a 5-point Likert scale ranging from 1 (none of the time) to 5 (all of the time). The total score calculated by summing responses across all items ranges from 14 to 70, with higher scores indicating greater positive mental well-being. The WEMWBS has demonstrated sound psychometric properties exhibiting acceptable validity and reliability [51, 53–55].

### Procedure

The study was conducted in compliance with the Declaration of Helsinki. Ethical approval was sought and obtained from the University of Nigeria Health Research Ethics Committee on certificate number- NHREC/05/01/2008B-FWA00002458-1RB00002323. Participants were recruited through community outreach. Three trained research assistants were deployed across the selected neighborhoods. They carried out door-to-door canvassing, participated in local community meetings, and visited churches as well as primary health care centers to disseminate information about the study. Older adults were approached directly and informed about the study objectives and procedures. Contact information was obtained from those who expressed interest, and follow-up reminders were provided via phone calls to encourage attendance at the screening sessions. Written informed consent was obtained from participants, and research assistants provided support with questionnaire administration when necessary. Participants were assured that their participation in the study was completely voluntary and that they could withdraw at any moment, for any reason, without their medical care or legal rights being jeopardized. To maintain consistency, data collection was completed in a single session per participant. To ensure data completeness, research assistants reviewed each questionnaire immediately after completion. Any missing or unclear responses were addressed on-site with the participants. As a result, the dataset had no missing values.

### Statistical analysis

All statistical analysis was performed using SPSS Software version 23 for Windows (IBM, Chicago, IL, USA). Descriptive statistics were used to summarize participant demographic characteristics, neighborhood characteristics, mental well-being and mobility. Prior to analysis, the normality of continuous variables was assessed using the shapiro-wilk test. The data met normality assumptions to a reasonable degree, supporting the use of pearson correlation and multiple linear regression. Pearson correlation coefficients assessed the relationships of neighborhood characteristics with mobility and mental well-being. Correlation strengths were interpreted as following [56]: ≤ 0.2: Weak, 0.3–0.5: Fair, 0.6–0.7: Moderate and ≥ 0.8: Strong. Multiple linear regression analysis was subsequently used to identify the predictors of mobility and mental well-being. To further explore the independent contribution of each environmental predictor, a stepwise regression was conducted, with variables entered sequentially to assess their incremental explanatory power. Significance level was set at 0.05.

## Results

A total number of 162 older adults participated in this study. The participants’ mean age was 67.30 ± 6.36. Table 1 presents the descriptive analysis of the domain and total scores of the NEWS using mean and standard deviation (Mean ± SD). Land use mix diversity domain had the highest mean score (88.42 ± 23.08) while, connectivity domain has the lowest mean score (8.60 ± 1.38). The total mean score of the NEWS was (235.57 ± 24.58).

**Table 1:** Domain and total scores of the neighborhood environment walkability scale among older adults in enugu, Nigeria (August 2023 – April 2024)

Domains of NEWS	Mean ± SD
Residential density	14.58 ± 3.19
Land use mix diversity	88.42 ± 23.08
Land-use mix	15.90 ± 2.87
Connectivity	8.60 ± 1.38
Infrastructure and safety	41.77 ± 7.74
Aesthetics	16.07 ± 3.86
Safety from traffic	16.36 ± 2.59
Safety from crime	9.12 ± 2.77
Environmental description	15.40 ± 2.98
Child’s safety	9.33 ± 2.38
Total	235.57 ± 24.58

The participants had a mean total score of 54.10 ± 9.86 and 44.85 ± 25.34 on the WEMWBS and LSQ respectively. On the WEMWBS, majority of the participants [63(77.8%)], had good psychological functioning while the rest (13.6% and 8.6%) had possible/mild depression and probable clinical depression respectively. Results of the Pearsons correlation test in Table 2 showed that residential density (*r*= -0.223, *p* = 0.004*), connectivity (*r* = 0.266, *p* = 0.001*) aesthetics (*r* = 0.212, *p* = 0.007*), safety from traffic (*r* = 0.214, *p* = 0.006*) all had a significant weak correlation with mobility. On the other hand, as shown in Table 2 land use mix diversity (*r*= -0.043, *p* = 0.589), land use mix (*r*= -0.125, *p* = 0.113), infrastructure and safety (*r* = 0.119, *p* = 0.133), safety from crime (*r* = 0.096, *p* = 0.223), additional environmental features (*r* = 0.117, *p* = 0.139), and child safety (*r*= -0.118, *p* = 0.134) all had a non-significant correlation with mobility.

**Table 2:** Pearsons correlation between mobility and neighborhood characteristics among older adults in enugu, Nigeria (August 2023 – April 2024)

	*R*	*P*
Residential density	-0.223	0.004*
Land use mix diversity	-0.043	0.589
Land-use mix	-0.125	0.113
Connectivity	0.266	0.001*
Infrastructure and safety	0.119	0.133
Aesthetics	0.212	0.007*
Safety from traffic	0.214	0.006*
Safety from crime	0.096	0.223
Environmental description	0.117	0.139
Child’s safety	-0.118	0.134

**Legend**: r = Pearson correlation coefficient (≤ 0.2: Weak, 0.3–0.5: Fair, 0.6–0.7: Moderate and ≥ 0.8: Strong); p = p value; *Significant at *p* < 0.05

Table 3 shows the results of multiple linear regression analysis to determine the neighborhood characteristics that predict mobility. The analysis showed a good model fit and was significant [F (10,151) = 7.389, *p* < 0.001, adjusted R^2^ = 0.28, R^2^ = 0.33]. The predictors/independent variables in the model accounted for 33% of the variance in mobility. Durbin Watson’s score was 2.066 indicating independence of observations in the model. Variance Inflation factor values were less than 10 while Tolerance values were greater than 0.20 indicating no multicollinearity in the model (Table 3). Residential density, land use mix, connectivity, aesthetics, safety from traffic, environmental description and child safety significantly predicted mobility (Table 5). Therefore, for every unit increase in residential density, land use mix, and child safety, mobility would likely decrease by 1.838, 1.394, 0.750 and 3.616 units respectively. More so, for every unit increase in connectivity, aesthetics, safety from traffic, and environmental description; mobility would increase by 8.077, 1.140, 1.555 and 1.666 units respectively.

**Table 3:** Predictors of mobility among older adults in enugu, Nigeria (August 2023 – April 2024)

Coefficients^a^									
Model	Unstandardized Coefficients		Standardized Coefficients	T	Sig.	95.0% Confidence Interval for B		Collinearity Statistics	
	B	Std. Error	Beta			Lower Bound	Upper Bound	Tolerance	VIF
(Constant)	-28.669	26.915		-1.065	0.289	-81.847	24.510		
Residential density	-1.838	0.568	− 0.231	-3.236	0.001	-2.960	− 0.716	0.870	1.149
Land use mix diversity	0.054	0.083	0.049	0.651	0.516	− 0.110	0.219	0.771	1.297
Land-use mix	-1.394	0.678	− 0.158	-2.056	0.042	-2.734	− 0.054	0.756	1.323
Connectivity	8.077	1.386	0.438	5.827	0.000	5.338	10.816	0.785	1.273
Infrastructure and safety	0.464	0.281	0.142	1.648	0.101	− 0.092	1.020	0.601	1.663
Aesthetics	1.140	0.503	0.174	2.268	0.025	0.147	2.134	0.760	1.316
Safety from traffic	1.555	0.760	0.159	2.045	0.043	0.053	3.057	0.734	1.363
Safety from crime	− 0.750	0.723	− 0.082	-1.038	0.301	-2.180	0.679	0.710	1.408
Environmental description	1.666	0.704	0.196	2.365	0.019	0.274	3.058	0.649	1.541
Child safety	-3.616	0.816	− 0.340	-4.432	0.000	-5.228	-2.004	0.756	1.323
Dependent Variable: mobility									

**Legend**: B = unstandardized regression coefficient; Std. Error = standard error of B; Beta = standardized regression coefficient; T = t-statistic; Sig. = probability value (p-value), with *p* < 0.05 considered statistically significant; 95% CI = 95% confidence interval for B; Tolerance and VIF = collinearity statistics (Tolerance < 0.20 and VIF > 10 indicate multicollinearity)

A stepwise multiple regression was subsequently conducted to determine the incremental contribution of key neighborhood variables in predicting mobility. While the initial regression model (Table 3) included all theoretically relevant variables, the stepwise approach iteratively retained only the most significant predictors, providing a more parsimonious model. This approach also helped to assess how much additional variance in mobility could be explained at each step of model building allowing an evaluation of their unique predictive value. It also provided a better understanding of the unique contribution of each environmental characteristic to mobility.

The model summary for the stepwise regression showed a progressive improvement in the model’s explanatory power across five steps (Table 4). Durbin Watson’s score for the model was 2.027 indicating independence of observations. The stepwise analysis yielded a final model (model 5) with five significant predictors; connectivity, residential density, aesthetics, child safety, and traffic safety (Table 3). The final model explained 27.5% of the variance in mobility scores (R² = 0.275, adjusted R² = 0.252), and was statistically significant (F(5, 156) = 11.83, *p* < 0.001). Although the stepwise regression model accounted for slightly less variance than the initial model that included all predictors, it provided a more parsimonious and interpretable framework. Importantly, it reaffirmed the predictive value of key neighborhood characteristics in explaining mobility among older adults. The consistency of core predictors across both models underscores the robustness of the findings. Notably, while land use mix and environmental description were statistically significant in the full model (Table 3), they were not retained in the final stepwise model (Table 4). This likely reflects the reduced unique contribution of these variables once more influential predictors such as connectivity, aesthetics, and traffic safety were accounted for. Their exclusion does not diminish their conceptual relevance; rather, it suggests that their effects may be partially mediated by or correlated with other environmental features captured in the final model.

**Table 4:** Model summary of Stepwise regression predicting mobility among older adults in enugu, Nigeria (August 2023 – April 2024)

Model	*R*	*R* Square	Adjusted *R* ^2^	Std. Error of the Estimate	Change Statistics					Durbin-Watson
					*R* Square Change	F Change	df1	df2	Sig. F Change	
1	0.266^a^	0.071	0.065	24.425	0.071	12.212	1	160	0.001	
2	0.379^b^	0.144	0.133	23.522	0.073	13.532	1	159	0.000	
3	0.436^c^	0.190	0.174	22.955	0.046	8.952	1	158	0.003	
4	0.474^d^	0.225	0.205	22.527	0.035	7.060	1	157	0.009	
5	0.524^e^	0.275	0.252	21.853	0.050	10.830	1	156	0.001	2.027

**Legend**: a. Predictors: (Constant), connectivity

b. Predictors: (Constant), connectivity, residential density

c. Predictors: (Constant), connectivity, residential density, aesthetics

d. Predictors: (Constant), connectivity, residential density, aesthetics, child safety

e. Predictors: (Constant), connectivity, residential density, aesthetics, child safety, safety from traffic

Dependent Variable: mobility

R = correlation coefficient; R² = proportion of variance in the dependent variable explained by the model; Adjusted R² = R² adjusted for the number of predictors in the model; Std. Error of the Estimate = standard deviation of the residuals; R² Change = change in explained variance with each step; F Change = change in F statistic for the step; df1, df2 = degrees of freedom; Sig. F Change = probability value for F change (*p* < 0.05 indicates significance); Durbin-Watson = test statistic for independence of residuals

Table 5 shows the coefficients for the final model, with connectivity emerging as the strongest positive predictor of mobility (β = 0.343, *p* < 0.001). For every unit increase in connectivity, aesthetics, and safety from traffic; mobility would increase by 6.323, 1.696 and 2.310 units respectively. Also, for every unit increase in residential density and child safety; mobility would decrease by 1.749 and 2.631 units.

**Table 5:** Model coefficients for final regression model (Model 5) predicting mobility among older adults in enugu, Nigeria (August 2023 – April 2024)

Model		Unstandardized Coefficients		Standardized Coefficients	t	Sig.	
		B	Std. Error	Beta			
5	(Constant)	-24.564	19.466		-1.262	0.209	
	connectivity	6.323	1.284	0.343	4.926	0.000	
	residential density	-1.749	0.564	0.220	-3.103	0.002	
	aesthetics	1.696	0.456	0.258	3.720	0.000	
	child safety	-2.631	0.765	0.247	-3.439	0.001	
	safety from traffic	2.310	0.702	0.237	3.291	0.001	

Legend: Dependent Variable: mobility; B = unstandardized regression coefficient; Std. Error = standard error of B; Beta = standardized regression coefficient, representing the relative strength of each predictor; t = t-statistic; Sig. = probability value (p-value), with p < 0.05 considered statistically significant

Table 6 shows that mental well-being had a non-significant weak correlation with residential density (*r*= -0.059, *p* = 0.45), land use mix diversity(*r*= -0.024, *p* = 0.764), connectivity (*r* = 0.025, *p* = 0.752), land use mix (*r*= -0.054, *p* = 0.498), infrastructure and safety (*r* = 0.078, *p* = 0.324), aesthetics (*r* = 0.113, *p* = 0.152), safety from traffic (*r*= -0.153, *p* = 0.051), safety from crime (*r* = 0.127, *p* = 0.107), environmental description(*r*= -0.042, *p* = 0.597), and child safety (*r*= -0.065, *p* = 0.413).

**Table 6:** Pearsons correlation between mental well-being and neighborhood characteristics among older adults in enugu, Nigeria (August 2023 – April 2024)

	*R*	*P*
Residential density	-0.059	0.45
Land use mix diversity	-0.024	0.764
Land-use mix	-0.054	0.498
Connectivity	0.025	0.752
Infrastructure and safety	0.078	0.324
Aesthetics	0.113	0.152
Safety from traffic	-0.153	0.051
Safety from crime	0.127	0.107
Environmental description	-0.042	0.597
Child’s safety	-0.065	0.413

**Legend**: r = Pearson correlation coefficient (≤ 0.2: Weak, 0.3–0.5: Fair, 0.6–0.7: Moderate and ≥ 0.8: Strong); p = p value; *Significant at *p* < 0.05

Table 7 presents the results of multiple linear regression analysis to determine the neighbourhood characteristics that predict mental well-being. The overall model was not significant [F (10,151) = 1.443, *p* = 0.167, adjusted R^2^ = 0.03, R^2^ = 0.09]. Residential density, land use mix diversity, land-use mix connectivity, infrastructure and safety, aesthetics, safety from traffic, safety from crime, environmental description and child safety were not significant predictors of mental well-being.

**Table 7:** Predictors of mental well-being among older adults in enugu, Nigeria (August 2023 – April 2024)

Coefficients^a^										
Model		Unstandardized Coefficients		Standardized Coefficients	T	Sig.	95.0% Confidence Interval for B		Collinearity Statistics	
		B	Std. Error	Beta			Lower Bound	Upper Bound	Tolerance	VIF
	(Constant)	68.009	12.207		5.571	0.000	43.891	92.128		
	Residential density	-0.328	0.258	-0.106	-1.273	0.205	-0.837	0.181	0.870	1.149
	Land use mix diversity	-0.020	0.038	-0.047	-0.526	0.600	-0.095	0.055	0.771	1.297
	Land-use mix	-0.272	0.308	-0.079	-0.885	0.378	-0.880	0.336	0.756	1.323
	Connectivity	0.265	0.629	0.037	0.421	0.674	-0.977	1.507	0.785	1.273
	Infrastructure and safety	0.034	0.128	0.027	0.269	0.789	-0.218	0.286	0.601	1.663
	Aesthetics	0.228	0.228	0.089	0.998	0.320	-0.223	0.678	0.760	1.316
	Safety from traffic	-0.887	0.345	-0.233	-2.573	0.011	-1.568	-0.206	0.734	1.363
	Safety from crime	0.654	0.328	0.184	1.994	0.048	0.006	1.302	0.710	1.408
	Environmental description	0.009	0.319	0.003	0.029	0.977	-0.622	0.640	0.649	1.541
	Child safety	-0.216	0.370	-0.052	-0.583	0.561	-0.947	0.515	0.756	1.323
	Dependent Variable: Mental well-being									

**Legend**: B = unstandardized regression coefficient; Std. Error = standard error of B; Beta = standardized regression coefficient; T = t-statistic; Sig. = probability value (p-value), with *p* < 0.05 considered statistically significant; 95% CI = 95% confidence interval for B; Tolerance and VIF = collinearity statistics (Tolerance < 0.20 and VIF > 10 indicate multicollinearity)

## Discussion

The findings of this study underscore the intricate relationships of mobility and mental well-being with neighborhood characteristics of older adults in Nigeria, offering insights that are vital for urban planning and public health interventions. Additionally, the study contributes significantly to the limited body of knowledge from sub-Saharan Africa. These findings are essential for bridging gaps in evidence, policy, and holistic management of aging populations in this region, where geriatric care is increasingly vital for enhancing quality of life and mitigating healthcare costs.

### Neighbourbood characteristics and mobility

In urban environments, mobility plays a critical role in fostering independence, social participation, and mental health among older adults [7, 21]. The study identified a negative relationship between residential density and mobility, indicating that higher density can hinder movement in older adults. This finding aligns with previous studies suggesting that overcrowded environments can discourage walking due to congestion, noise, and lack of personal space [57, 58]. Urban areas face challenges posed by overcrowded neighborhoods, including limited open spaces and pedestrian pathways, which restrict movement [60, 61]. In densely populated Nigerian urban centers, rapid urbanization often results in poorly designed high-density residential areas with limited pedestrian pathways and inadequate infrastructure, thereby impeding mobility [62]. However, it is important to note that high residential density is not necessarily disadvantageous. In well-organized urban areas, higher density can facilitate access to essential services, amenities, and public transport, thereby reducing the need for long-distance travel and encouraging regular activity and social engagement [2, 18]. While poorly planned high-density neighborhoods may pose barriers to mobility due to congestion and inadequate infrastructure, better-designed environments that include safe pedestrian routes, green spaces, and conveniently located services can help to support mobility and improve access. Therefore, policies promoting adequate spacing, walkable pathways, and green spaces in high-density areas could mitigate these barriers and enhance mobility.

Contrary to expectations, land-use mix diversity showed no significant relationship with mobility in this study and was not a significant predictor. This finding contrasts with previous research suggesting that diverse land use promotes walking by providing proximate destinations such as shops, parks, and healthcare facilities [63, 64]. In many Nigerian urban areas, however, land use planning is often inconsistent, with commercial, residential, and industrial zones intermingled haphazardly [65]. This disorganization may negate the functional benefits typically associated with land use mix diversity. Although this variable had the highest mean score among the neighbourhood characteristics in the current study, its lack of correlation with mobility could also stem from poor accessibility or unsafe routes issues which are prevalent in Nigerian cities [5, 66]. Achieving land use diversity alone is insufficient if essential services are inaccessible or if the quality of infrastructure remains inadequate. Policies that address these challenges are crucial. To enhance mobility, deliberate zoning strategies and the development of accessible, mixed-use areas should be prioritized. Such policies could ensure that essential services and recreational spaces are within safe walking distances, thereby maximizing the benefits of land use diversity. Additionally, improving pedestrian infrastructure and safety is essential to fully realize the mobility potential of such developments.

Similarly, land use mix was neither significantly associated with mobility nor a predictor of it. While mixed-use neighborhoods theoretically encourage walking by placing destinations within easy reach, the lack of well-maintained sidewalks, inadequate pedestrian crossings, and unsafe environments significantly diminishes the usability of these spaces [67, 68]. These factors pose barriers and are obstacles to leveraging mixed-use developments for improved mobility, particularly for vulnerable groups such as older adults [36, 68]. To address these challenges, urban planning in Nigeria should prioritize creating safe, accessible, and pedestrian-friendly mixed-use neighborhoods. Investments in infrastructure, such as well-maintained sidewalks and adequate crosswalks, are essential to maximize the potential of mixed-use developments to support mobility and active living.

Connectivity was found to be significantly associated with mobility in the current study with it emerging as a positive predictor. The ability to navigate neighborhoods effectively is presumed to be closely influenced by the characteristics of the built environment. Connectivity, characterized by well-designed street networks with shorter block lengths and higher intersection density, enhances accessibility by providing easier and more direct routes to destinations [18, 63]. The current study findings on the relationship between connectivity and mobility aligns with global research emphasizing that connected neighborhoods encourage walking by reducing travel distances and increasing accessibility to essential services and recreational areas [18, 19]. However, in the Nigerian context, poor road infrastructure such as pothole-ridden surfaces, inadequate drainage, and unpaved roads significantly impairs neighborhood connectivity [5, 62]. In many urban areas, especially informal settlements, the lack of structured road layouts, unmarked pathways, and congested alleys makes navigation difficult for older adults [36, 62, 65]. These environments often lack designated pedestrian routes, which discourages walking and increases dependence on motorized transport. Furthermore, informal settlements that emerge without adherence to zoning regulations often develop organically with limited accessibility to public services and essential facilities [37, 38, 65]. Such unplanned developments hinder route options and complicate direct travel between locations, further restricting mobility. The challenges faced by Nigerian cities stem from rapid urbanization, which often outpaces infrastructure development, leading to haphazard urban planning and poorly connected neighborhoods [5, 62, 65]. This mismatch between population growth and infrastructure provision disproportionately affects older adults, whose ability to move freely depends heavily on neighborhood connectivity. This disorganization reduce access to healthcare, social opportunities, and physical activity spaces, exacerbating risks of isolation and mobility decline [8, 11]. Despite these barriers, connectivity offers a promising path to improving mobility and active aging. Urban planning efforts must prioritize the redesign of poorly connected areas, particularly in informal settlements, by integrating formal road systems and improving accessibility features. Investments in basic infrastructure such as paving roads, installing street signage, and constructing safe pedestrian crossings are essential. Also, prioritizing accessible environments such as well-maintained sidewalks, safe crossings, and regulated vending zones can enhance independence, reduce sedentary behavior, and promote active lifestyles among older adults.

Infrastructure and safety did not show a significant correlation with mobility, aligning with studies that emphasize the need for accessible and well-maintained environments to support mobility [69, 70]. However, this does not negate their importance. Although no direct relationship was observed, regression analysis identified infrastructure and safety as significant predictors of mobility. Features like well-lit streets, pedestrian crossings, and reliable public transport are essential for creating age-friendly environments that foster mobility and independence [69, 70]. In Nigeria, infrastructural deficits, unregulated street vending, and traffic hazards discourage walking, especially among older adults [5, 66, 70, 71]. Investments in age-friendly infrastructure, regulation of street vending, traffic management and safety measures are critical. These efforts, when combined with urban planning that prioritizes walkability, can reduce sedentary behavior, enhance physical activity, and improve overall well-being [2, 15]. Such measures not only promote active aging but also improve the quality of life for older adults by fostering independence and community engagement [1, 28].

Neighborhood aesthetics, including green spaces, tree-lined streets, and well-maintained public areas, play a vital role in promoting mobility particularly among older adults [28, 72]. A significant positive association between aesthetics and mobility in this current study underscores the impact of visually appealing environments in encouraging walking and outdoor activity [8, 11]. Each unit increase in aesthetics was associated with greater mobility among participants. Despite these benefits, many Nigerian cities face challenges such as urban decay, litter, and a lack of greenery, which limit the positive effects of aesthetics [5, 73, 75]. Beautification projects often target affluent areas, leaving underserved neighborhoods neglected [5, 74]. This inequity highlights the need for inclusive urban planning to create equitable access to mobility-enhancing environments. Integrating greening initiatives into underserved areas can bridge this gap, promoting equity and improving mobility. Given Nigeria’s urbanization and aging population, addressing infrastructure disparities is essential for active aging.

Perceived safety from traffic was significantly associated with mobility and positively predicted mobility in the current study, underscoring the importance of secure environments for encouraging outdoor activities [72, 75]. However, poor pedestrian infrastructure and inadequate traffic regulation in Nigerian cities increase the risk of traffic-related injuries, discouraging walking [74, 76]. Enhancing pedestrian infrastructure, implementing traffic-calming measures, and strengthening road safety regulations could improve safety and mobility. Safety from crime showed no significant association with mobility and was not a predictor in the current study. While perceived safety is a critical determinant of walking behavior [77], the findings suggest that poor infrastructure and environmental harzards may overshadow crime concerns in the Nigerian context. However, this does not diminish the importance of addressing safety concerns as part of a comprehensive approach to improving mobility. Crime-related safety concerns often deter outdoor activities, limiting physical and social engagement [78]. In Nigeria, where informal social structures such as community surveillance and high levels of social interaction exist [79, 80], these factors may mitigate the negative impact of crime on mobility. Nevertheless, enhancing neighborhood security through better street lighting, community policing, and crime prevention strategies could further encourage outdoor mobility and improve the quality of life for older adults. Environments perceived as safe from crime and conducive to walking reduce sedentary behavior and encourage outdoor activities, which are essential for maintaining optimal health [15, 28].

Environmental description did not show a significant correlation with mobility but emerged as a significant predictor in this study. This finding suggests that perception of the neighborhood environment could influence mobility. Such improvements align with existing research that underscores the importance of creating safe, walkable, and accessible neighborhoods to support active aging [2, 15]. By addressing the unique challenges posed by rapid urbanization and population aging in Nigeria, these findings emphasize the critical need for urban planning and policy interventions that promote active lifestyle, social engagement, and overall well-being among older adults.

The observed negative correlation between perceptions of child safety and mobility among older adults in Nigeria suggests a paradox: higher perceived child safety is associated with reduced mobility in this population. This counterintuitive finding may reflect prevailing cultural norms in Nigeria, where older adults frequently assume substantial caregiving responsibilities, particularly for grandchildren. These caregiving duties can constrain their mobility, as time and energy are redirected from personal activities to child supervision A study by Aransiola et al. [81] and Ezulike et al. [82] highlights that caregiving roles for the elderly is associated with increased stress and reduced personal time, which can further impede mobility. Compounding this issue, Nigeria’s rapid urbanization has introduced significant infrastructural challenges that affect the safety and mobility of both children and older adults. Inadequate pedestrian infrastructure, traffic risks, and poor urban planning contribute to environments that hinder movement for these vulnerable groups. Implementing urban safety measures such as designated pedestrian zones, effective traffic management, and well-maintained walkways could significantly enhance mobility across all age groups. The World Health Organization underscores the importance of thoughtfully designed urban environments in promoting independence and fostering social connectedness among older adults. Addressing these infrastructural deficits while considering cultural caregiving expectations may offer a pathway to improving mobility outcomes for Nigeria’s aging population.

Futhermore, the exclusion of land use mix and environmental description from the final hierarchical model, despite their significance in the initial regression, is noteworthy. This likely reflects shared variance with more influential predictors, such as connectivity and aesthetics, which more directly capture the elements of the neighborhood that facilitate or constrain mobility. Stepwise regression emphasizes variables with unique explanatory power, and as such, land use mix and environmental description may still play indirect or synergistic roles in shaping mobility behavior. Their theoretical importance in urban and aging literature, particularly in studies of walkability and perceived environmental quality, remains strong and warrants further exploration using mediation or interaction models.

### Neighbourhood characteristics and mental well-being

Walkable neighborhoods with accessible pathways and proximity to amenities foster physical activity, social engagement, and mental health benefits [2, 15]. The rapid urbanization and population aging in Nigeria present unique challenges that complicate the relationship between walkability and mental well-being. As highlighted in the introduction, Nigeria’s urban environments are often characterized by unplanned settlements, inadequate infrastructure, and socio-economic inequalities, which exacerbate vulnerabilities among older adults [5, 37]. These contextual realities may limit the mental health benefits of walkability typically observed in high-income settings.

Walkability is widely recognized as a determinant of physical and mental health in other contexts [18, 19]. Walkable neighborhoods are often praised for promoting physical activity, social interaction, and reduced sedentary behavior, all of which contribute to mental well-being [15, 17]. However, our findings challenge these established assumptions by revealing non-significant correlations between mental well-being and key neighborhood characteristics. Also, neighborhood characteristics were not significant predictors of mental well-being. These results underscore the need to contextualize the relationship between neighborhood characteristics and mental health, particularly in low and middle income countries like Nigeria, where unique socio-cultural and infrastructural limitations may mediate these associations. The non-significant relationships observed in this study suggest that the presumed benefits of walkability may not automatically translate into improved mental health outcomes for older adults in Nigeria. This aligns with studies highlighting the importance of addressing contextual factors such as urban planning deficiencies, perceptions of safety, and infrastructural quality [5, 60, 62]. For example, unplanned settlements and high residential density without adequate amenities or pedestrian infrastructure may negate the advantages of walkable neighborhoods, particularly in rapidly urbanizing cities. High residential density, a core component of walkability, has been associated with greater access to amenities and social opportunities in high-income countries [83, 84]. However, in Nigeria’s urban settings, the absence of adequate pedestrian pathways, green spaces, and essential services may negate these benefits [73, 74]. This aligns with prior research showing that without supporting infrastructure, high-density living can become a source of stress rather than a facilitator of well-being [2].

Similarly, the non-significant relationships between mental well-being and variables such as land-use mix diversity, land-use mix, and connectivity observed in this study suggest that accessibility to amenities and neighborhood interconnectedness alone may not be sufficient to promote mental well-being in Nigerian urban environments. The lack of comprehensive urban planning, inadequate infrastructure, and socio-economic inequalities exacerbate vulnerabilities among older adults, affecting both their physical mobility and psychological health [5, 37]. Thus, in contexts like Nigeria, where these issues are pervasive, these findings underscore the urgent need for urban planning approaches that prioritize both the physical and psychological dimensions of walkability.

Neighborhood safety, a critical factor influencing mental well-being in existing literature [15, 85], also did not show significant correlations in this study. This discrepancy may arise from the interplay between actual crime rates, perceptions of safety, and the broader socio-economic disparities prevalent in Nigerian urban areas. For instance, Yeon et al. [86] demonstrated that older adults’ psychological health is significantly shaped by how safe they perceive their neighborhoods to be, especially among those with functional limitations. Perceptions of safety whether shaped by crime rates or traffic hazards remain critical in determining the extent to which older adults engage with their neighborhoods. However, in settings characterized by systemic insecurity and inadequate policing, such as Nigeria, these perceptions may be shaped more by societal conditions than immediate neighborhood environments [5, 37]. A study conducted in Enugu State, Nigeria, reported that perceived neighborhood safety significantly influenced physical activity levels and social participation among older adults, highlighting the profound impact of safety perceptions on daily functioning and overall well-being [87].

Fear of crime has previously been shown to limit mobility, discourage outdoor physical activity, and reduce opportunities for social interaction, all of which are essential for mental health [88]. While this current study did not find significant associations between neighborhood safety and mental well-being, the findings reinforce the need for targeted interventions to enhance perceived safety. Strategies such as improved street lighting, community policing, and neighborhood-level social cohesion initiatives could help mitigate the adverse effects of insecurity on older adults’ mental health. Connectivity, which has the potential to reduce social isolation and enhance mobility in well-designed urban environments [18, 19], did not yield these benefits in Nigerian urban cities, where traffic hazards and poorly maintained pathways likely undermine its advantages. Investments in pedestrian-friendly.infrastructure and the integration of green spaces could help address these deficits, as these features have been shown to enhance both physical and mental well-being in older populations globally [19, 64]. In Nigeria specifically, neighborhood connectivity, combined with physical activity, has been shown to plays a significant role in the mental well-being of older adults in Nigerian urban settings [88]. Ultimately, for neighborhood connectivity to positively influence the mental well-being of older adults, the environment must not only be well-connected, but must also feel safe and be supported by adequate infrastructure that encourages active and confident engagement with the neighborhood.

Neighborhood aesthetics, encompassing the visual appeal of the environment, presence of green spaces, and cleanliness, are often cited as factors promoting mental well-being by encouraging outdoor activity and fostering a sense of place [19, 28]. However, the non-significant relationship between aesthetics and mental well-being in this study suggests that in Nigerian urban environments, aesthetic considerations may be secondary to more pressing concerns. Poor urban planning, inadequate waste management systems, and limited access to greenery in many Nigerian cities likely diminish the role of aesthetics in influencing mental health outcomes [73].

Traffic safety has been linked to mental well-being in studies conducted in well-planned urban environments, where reduced traffic hazards promote outdoor mobility and social interactions [15, 64]. Yet, the absence of significant associations in this study indicates that systemic infrastructure deficits and unsafe pedestrian pathways may limit the relevance of traffic safety in Nigerian contexts. Moreover, traffic safety alone may not compensate for broader environmental barriers that deter older adults from engaging in physical and social activities.

Environmental description, which captures overall perceptions of the neighborhood’s physical conditions, did not significantly correlate with mental well-being in this study. This finding contrasts with evidence from high-income countries where favorable environmental perceptions are associated with reduced stress and improved psychological health [23, 27]. In Nigerian cities, issues such as overcrowding, unplanned settlements, and environmental degradation [62] may contribute to negative perceptions potentially masking any positive effects on mental health.

Lastly, child safety, often used as a proxy for neighborhood social cohesion and collective efficacy, has been positively associated with mental well-being in some contexts, as it reflects the perceived safety and community support of the environment [2, 88]. However, in this study, child safety showed no significant relationship with mental well-being. Broader societal challenges in Nigeria, including high crime rates and systemic insecurity, may diminish perceptions of safety regardless of specific neighborhood conditions [5, 37].

This study is not without limitations. The cross-sectional design precludes causal inferences. Also, the reliance on self-reported measures introduces the potential for response bias, such as social desirability bias. To mitigate this, the authors ensured participant anonymity and confidentiality. Residential density was used to guide neighborhood stratification and ensure sampling diversity. While this approach helped represent a range of urban environments, it does not fully capture the multidimensional nature of walkability. Prior studies in Enugu have shown that walkability scores may overlap across density categories, which could affect the interpretation of neighborhood characteristics. Although residential density was not treated as an exposure variable in this analysis, future studies may benefit from stratifying neighborhoods using composite walkability indices to better reflect the complexity of the built environment.

Additionally, overlapping walkability characteristics across different density categories may have limited the ability to detect associations, particularly in relation to mental well-being. Future studies should consider using composite walkability indices to classify neighborhoods more precisely. In addition, adopting multilevel analytical designs would allow researchers to account for the nested structure of data, where individuals are clustered within neighborhoods, and to distinguish more clearly between individual-level and neighborhood-level influences on mobility and mental well-being.

### Implications for policy and urban design

The findings of this study on neighborhood walkability, mobility, and mental well-being among older adults highlight critical challenges and implications for urban planning and policy development in Nigeria and sub-Saharan Africa. As urbanization accelerates in Nigeria, particularly in rapidly growing cities like Enugu, Lagos and Abuja, the need to create age-friendly, walkable environments becomes imperative to address the mobility and well-being of older adults. These recommendations are based on the Ecological Model by Sallis et al. [89], which highlights the interaction between personal, environmental, and policy-related factors in influencing health outcomes. The model underscores the importance of addressing both physical and psychological needs in designing supportive urban environments for older populations. The following prioritized policy and design implications are proposed based on the study’s findings:

i. Promoting walkability through improved infrastructure (Medium to long term priority).

Policymakers should prioritize investments in pedestrian-friendly infrastructure, such as well-maintained sidewalks with sufficient width and accessibility features, safe pedestrian crossings, traffic calming measures, age-friendly public spaces including green spaces and recreational areas, to encourage physical activity and social engagement.

ii. Addressing residential density challenges (Medium term priority).

Urban planners should prioritize redesigning high-density areas to include adequate open spaces, pedestrian pathways, and green areas to promote ease of movement. Additionally, implementing zoning regulations is essential to ensure balanced land use that seamlessly integrates residential, commercial, and recreational spaces, thereby preventing overcrowding and fostering a more walkable environment. The redevelopment strategy in parts of Lagos Mainland, Nigeria may serve as a reference for addressing density while maintaining accessibility.

iii. Enhancing Connectivity (Long term priority).

Urban design strategies should focus on creating shorter block lengths and increasing intersection densities, thereby improving route options for pedestrians. Upgrading road networks and regulating street vending particularly in informal settlements are also vital to minimize obstructions and improve pedestrian access.

iv. Improving Perceived and Actual Safety (High/Immediate Priority).

While perceived safety from crime showed no significant impact on mobility, traffic safety concerns were prominent. Policymakers should immediately prioritize enforcing traffic regulations and speed limits especially in high traffic areas to mitigate pedestrian hazards. Additionally, improving street lighting, promoting community policing, and enhancing neighborhood security measures can foster a sense of safety.

v. Integrating Aesthetic Improvements (Medium-term priority).

The strong correlation between neighborhood aesthetics and mobility underscores the need for visually appealing environments. Urban beautification efforts should include tree planting, street greening, and public art installations. Efforts should focus on equitable distribution, particularly in underserved communities.

vi. Rethinking Land Use and Accessibility (Long term priority).

The lack of a significant relationship between land-use mix diversity and mobility reveals gaps in accessibility and infrastructure quality. Policies should focus on clustering essential services such as markets, clinics, and social centers within safe walking distances. Furthermore, targeted infrastructure upgrades should enhance access to amenities, especially in mixed-use developments.

vii. Creating Inclusive Policies for Vulnerable Groups (Medium term priority).

Older adults with functional impairments face distinct challenges in urban environments. To address these, interventions should include the installation of assistive features such as ramps, handrails, and elevators in public spaces. Additionally, community programs that encourage social interaction and active aging are necessary, especially in high-density or underserved neighborhoods.

viii. Bridging the Gap Between Mobility and Mental Well-Being (Cross-cutting priority).

The absence of significant associations between neighborhood characteristics and mental well-being in this study underscores the need for holistic approaches that integrate physical and psychological dimensions. This suggests that while the built environment plays a crucial role in fostering mobility, additional factors must be considered to enhance overall well-being. Based on the ecological model [89], interventions should integrate a comprehensive approach that incoporates physical, social, and psychological dimensions within urban planning and policy frameworks.

Policy makers should:

- Address Broader Societal Issues.

Policymakers must recognize that socio-economic inequalities, urban planning deficiencies, and resource disparities critically impact mental well-being. Narrowing income gaps and improving access to quality housing, healthcare, and education can provide a strong foundation for better mental health. Investments in underprivileged neighborhoods can enhance equity and inclusivity, reducing stressors linked to poor living conditions. Additionally, addressing urban planning shortcomings by ensuring equitable access to essential services and creating inclusive public spaces can foster social connection and mental resilience. By prioritizing these systemic changes, policymakers can create environments that actively support mental well-being.

b. Implement mental health programs within communities to complement physical mobility initiatives.

While urban mobility initiatives enhance physical environments, integrating mental health-focused programs is essential for holistic community well-being. Accessible neighborhood mental health services, such as counseling centers and support groups, can address psychological stressors when embedded in shared spaces like primary health care centers or recreational parks. Community-driven activities, like festivals and exercise groups, promote social cohesion and reduce isolation, fostering resilience. Additionally, awareness campaigns and stress management programs can combat stigma and empower individuals to seek support, creating safer, more mentally supportive communities.

By addressing these policy and design implications in a prioritized and context-specific manner, stakeholders can create urban environments that not only enhance mobility but also promote overall well-being among older adults. These efforts will ensure that older adults remain socially engaged, physically active, and mentally resilient in their communities.

### Future directions

The discrepancies between our findings and those of previous studies highlight the importance of further research to identify the mechanisms through which neighborhood characteristics influence mental well-being in low-income countries. Future research should adopt longitudinal approaches to better understand the dynamic relationships between neighborhood environments, mobility, and mental well-being. In particular, studies should consider mobility as a mediator or covariate in regression models exploring mental well-being, as this may provide additional insights into the pathways through which neighborhood characteristics exert their influence.

While the study primarily focused on self-reported perceptions to capture the lived experiences and contextual realities of older adults in Nigerian urban settings, we recognize that incorporating objective measures could offer deeper insights into how these features influence mobility and mental well-being. This remains a crucial area for future investigation.

Additionally, qualitative studies could provide deeper insights into older adults’ perceptions of their environments and how these perceptions shape their mobility and mental health. Moreover, future studies would benefit from incorporating key sociodemographic variables such as gender, income, and education to provide a more comprehensive understanding of the complex interplay between neighborhood environment, individual characteristics, and mental well-being. Integrating these factors will contribute to a more nuanced understanding of aging in urban Nigerian settings and help guide the development of more targeted and inclusive interventions.

## Conclusions

This study provides novel insights into the relationship of neighborhood characteristics with mobility and mental well-being of older adults in urban Nigeria, a context that remains underexplored in existing literature. Specifically, the findings reveal that residential density, connectivity, aesthetics, and safety from traffic were significantly related with the mobility of older adults. Furthermore, residential density, land use mix, connectivity, aesthetics, safety from traffic, environmental description, and child safety emerged as significant predictors of mobility. Conversely, no significant relationship was found between neighborhood characteristics and the mental well-being of older adults. This discrepancy highlights the intricate interplay of environmental, social, and cultural factors shaping mental well-being in rapidly urbanizing Nigerian settings.

The study makes a significant contribution by offering a context-specific perspective that underscores the importance of designing urban environments that accommodate the diverse needs of aging individuals. It emphasizes the urgent need for urban planners and policymakers to adopt context-sensitive approaches in prioritizing age-friendly environments such as pedestrian infrastructure, regulated traffic systems, and accessible green spaces to mitigate barriers to mobility while supporting the broader well-being of aging populations. By integrating these insights into urban planning and policy frameworks, stakeholders can more effectively meet the needs of older adults and enhance their quality of life.

Additionally, the study suggests the importance of tailored environmental interventions for older adults with and without functional limitations. Physically impaired older adults are particularly vulnerable to the adverse effects of poorly designed neighborhood environments and require targeted strategies to address their unique needs. Initiatives focused on improving neighborhood connectivity, ensuring pedestrian safety, and enhancing aesthetic appeal are crucial for enhancing mobility, promoting active aging, and ultimately enhancing the physical and mental well-being of this vulnerable population. By integrating these evidence-based insights into planning and policy frameworks, stakeholders can contribute meaningfully to building inclusive, supportive, and health-promoting urban environments for Nigeria’s growing aging population.

## Data Availability

The datasets used and/or analysed during the current study are available from the corresponding author on reasonable request.

## Data availability

The datasets used and/or analysed during the current study are available from the corresponding author on reasonable request.

## Abbreviations

LGA: Local Government Area
NEWS: Neighborhood Environment Walkability Scale
LSQ: Life Space Questionnaire
WEMWBS: Warwick Edinburgh Mental Well-being Scale
